# TRAF6 Suppresses the Development of Pulmonary Fibrosis by Attenuating the Activation of Fibroblasts

**DOI:** 10.3389/fphar.2022.911945

**Published:** 2022-05-20

**Authors:** Jiali Min, Qiao Li, Suosi Liu, Qianrong Wang, Min Yin, Yan Zhang, Jun Yan, Bing Cui, Shanshan Liu

**Affiliations:** ^1^ National Clinical Research Center for Metabolic Diseases, Key Laboratory of Diabetes Immunology, Ministry of Education, Department of Metabolism and Endocrinology, The Second Xiangya Hospital of Central South University, Changsha, China; ^2^ Beijing Institute of Brain Disorders, Capital Medical University, Beijing, China; ^3^ State Key Laboratory of Bioactive Substance and Function of Natural Medicines, Institute of Materia Medica, Chinese Academy of Medical Sciences and Peking Union Medical College, Beijing, China

**Keywords:** pulmonary fibrosis, TRAF6, lung fibroblast, TRIB3, ubiquitination, protein degradation, Wnt3a

## Abstract

Pulmonary fibrosis (PF) has a high mortality rate, and its pathogenesis is unknown. TNF receptor-associated factor 6 (TRAF6), a signal transducer for inflammatory signaling, plays crucial roles in the pathogenesis of immune diseases. However, its function in PF remains unknown. Herein, we demonstrated that lungs from mice with bleomycin (BLM)-induced PF were characterized by decreased expression of TRAF6 in lung fibroblasts. Enhancing TRAF6 expression protected mice from BLM-induced PF coupled with a significant reduction in fibroblast differentiation. Furthermore, we demonstrated that overexpression of TRAF6 reversed the activation of myofibroblasts from PF mice by reducing the expression of Wnt3a and subsequently suppressing Wnt/β-catenin signaling. Additionally, the abundance of Tribbles pseudokinase 3 (TRIB3), a stress sensor, was negatively correlated with the abundance of TRAF6 in lung fibroblasts. TRIB3 overexpression decreased TRAF6 abundance by reducing TRAF6 stability in lung fibroblasts during PF. Mechanistic studies revealed that TRIB3 bound to TRAF6 and accelerated basal TRAF6 ubiquitination and degradation. Collectively, our data indicate that reduced TRAF6 expression in fibroblasts is essential for the progression of PF, and therefore, genetically increasing TRAF6 expression or disrupting the TRIB3-TRAF6 interaction could be potential therapeutic strategies for fibroproliferative lung diseases in clinical settings.

## Introduction

Pulmonary fibrosis (PF) is a type of chronic, progressive, and irreversible fibrotic lung disease characterized by an excess of extracellular matrix (ECM), destruction of normal lung architecture, and ultimately, respiratory failure (Sgalla et al., 2019; George et al., 2020). To date, only two therapies, pirfenidone and nintedanib, have been approved for use in PF. However, neither treatment is known to be curative. Lung transplantation remains the final and life-saving therapeutic option for PF. The lack of a successful treatment of PF is due to a lack of understanding of the underlying cellular and molecular mechanisms. Therefore, an in-depth investigation of the molecular pathogenesis of PF is urgently needed.

Although the cause of PF remains unclear, existing evidence suggests that lung fibroblasts play a crucial role in the pathogenesis of PF (Hinz and Lagares, 2020; Liu G. et al., 2021). Upon stimulation of fibrotic factors, such as chemokine (C-C motif) ligand 1 (CCL1), transforming growth factor-β1 (TGF-β1), and platelet-derived growth factor (PDGF), fibroblasts differentiate into myofibroblasts, which secrete excessive amounts of ECM proteins, including type I collagen, and fibronectin (Lo Re et al., 2011; Frangogiannis, 2020; Liu S. S. et al., 2021). Increased accumulation of ECM obliterates functional alveolar units, thereby decreasing gas exchange and reducing lung function. It is critically important to identify the key mediators contributing to the activation of myofibroblasts during PF development.

TRAF6 (TNF receptor-associated factor 6), a member of the TRAF protein family, plays a pivotal role in immune signal transduction pathways triggered by members of the TNF receptor superfamily and the IL-1 receptor superfamily (Cao et al., 1996; Walsh et al., 2015). By acting as a ubiquitin ligase (E3) catalyzing the formation of Lys63-linked polyubiquitin on itself and its substrates, TRAF6 participates in many biological processes, such as the operation of the innate immune system, lymph node organogenesis, and osteoclast formation (Naito et al., 1999; Wu and Arron, 2003; Yang et al., 2009). TRAF6 contains a RING-finger domain in the N-terminus, which is responsible for its E3 ligase activity, followed by several zinc-finger domains and a conserved C-terminal TRAF domain that enables interaction with receptors and adaptor proteins. Recent studies have revealed the role of TRAF6 in fibrosis (Wang et al., 2020; Zhang et al., 2021). TRAF6 overexpression in hepatocytes promotes ubiquitination-dependent activation of ASK1, contributing to the pathogenesis of hepatic fibrosis during the progression of nonalcoholic steatohepatitis (NASH) (Wang et al., 2020). However, it remains unclear whether TRAF6 plays any important roles in the pathogenesis of PF.

In the present study, we observed reduced protein levels of TRAF6 in the lungs of PF mice, supporting a role for TRAF6 in PF development. Using the classical bleomycin (BLM)-induced PF mouse model, we found that overexpression of TRAF6 reduced BLM-induced PF changes. Using gain- and loss-of-function approaches *in vitro*, we demonstrated that the downregulation of TRAF6 expression is induced by the upregulation of expression of TRIB3 in lung fibroblasts during chronic lung injury. Furthermore, the loss of TRAF6 aggravated the progression of PF by acting as a novel negative regulator of Wnt3a, thereby activating lung fibroblasts into myofibroblasts. Collectively, our findings revealed a critical role for TRAF6 in suppressing the activation of lung fibroblasts and preventing the development of PF.

## Materials and Methods

### Antibodies and Reagents

Antibodies against TRAF6 and TRIB3 were purchased from Abcam (Cambridge, MA, United States); antibodies against Axin 2, c-Myc, and cyclin D1 were obtained from Cell Signaling Technology (Danvers, MA, United States); an antibody against α-SMA was purchased from BOSTER (Wuhan, China); and antibodies against DDK, Myc and HA were obtained from MBL BIOTECH (Beijing, China). CoraLite® 594-phalloidin was obtained from Proteintech (Wuhan, China). CHX was purchased from Sigma (St Louis, MO, United States). Bleomycin (BLM), MG132, and 3-Methyladenine (3-MA) were obtained from MCE (Shanghai, China).

### Bleomycin (BLM) Induction of Pulmonary Fibrosis

C57BL/6J male mice (8 weeks old) were purchased from SJA Laboratory Animal Co., Ltd. (Hunan, China). The mouse PF model was induced as previously reported (Liu et al., 2019). In brief, mice were anesthetized with 400 mg/kg avertin (Sigma–Aldrich) via intraperitoneal injection and then treated with 1 mg/kg BLM in 50 μl PBS via intratracheal injection. This was conducted 6 times, with 14 days between each challenge. Mice were sacrificed by excessive anesthesia at Day 40 after the last BLM challenge, and then lungs were obtained for immunoblotting and RT–PCR analysis.

### Lentiviral Treatment in Vivo

For lentivirus administration, lentiviruses (5 × 10^7^ I.U.) overexpressing *Traf6* in 50 μl of PBS were administered to mice via intratracheal instillation for a total of two treatments at 2-weeks intervals beginning on Day 10 after the last BLM administration.

### Isolation of Lung Fibroblasts

Lung fibroblasts were obtained from mice as reported previously (Liu S. S. et al., 2021). The chests of mice sacrificed by excessive anesthesia were cut open, and the lungs were removed, rinsed with sterile PBS and cut into 1 mm^3^ pieces in culture medium. After centrifugation at 1,500 rpm for 10 min, the tissue suspension was suspended in DMEM containing 15% FBS and spread evenly in a 10-cm dish. After 5–7 days of culture, the adherent fibroblasts were harvested for passage or for assays.

### Cell Lines

MRC5 fibroblasts were cultured in minimal essential medium supplemented with 10% FCS, penicillin, streptomycin, and non-essential amino acid (NEAA) at 37°C in a humidified 5% CO_2_ incubator.

### Cell Transfection

Plasmids were transfected into cells with Lipofectamine 3,000 (Invitrogen) according to the manufacturer’s instructions. siRNAs were transfected using Lipofectamine RNAiMAX (Invitrogen) according to the manufacturer’s instructions.

### Invasion Assays

Fibroblasts were cultured in serum-free DMEM for 24 h prior to cell invasion assays. Transwell invasion assays were performed using Transwell chambers with filter membranes of 8 μm pore size (Corning). The lower surface of the chambers was precoated with 10 μg ml^−1^ fibronectin, and the upper chambers were coated with BD Matrigel™ Basement Membrane Matrix (40 μl per well). Then, the chambers were inserted into 24-well culture plates. Single-cell suspensions were seeded into the upper chamber (1 × 10^4^ cells per well in 0.4% FBS in DMEM), and the bottom chamber contained DMEM with 10% FBS. After 12 h, the medium was removed, and noninvasive cells were removed using cotton swabs. The invaded cells were fixed with 4% paraformaldehyde in PBS and stained with crystal violet staining solution. Invasive cells from 3 nonoverlapping fields of each membrane were imaged and counted using brightfield microscopy under 40x magnification.

### RNA Extraction and Real-Time Polymerase Chain Reaction

Total RNA was isolated from cells and tissues using the Eastep® Super Total RNA Extraction Kit (Promega) according to the manufacturer’s instructions. RNA was quantified using a NanoDrop spectrophotometer. Reverse transcription was carried out with 1 mg of purified RNA using M-MLV reverse transcriptase (Transgen Biotech). Quantitative real-time PCR (qRT–PCR) was performed using the KAPA SYBR FAST RT–PCR Master Mix (2×) Kit (Kappa Biosystem, United States) and conducted by a LineGene 9,620 apparatus (Bioer). All primer details are listed in Supplementary Table S1.

### Immunoblot

Tissues and cultured cells were lysed in RIPA buffer (Beyotime) containing protein inhibitors (Selleck). The protein concentration was determined by a BCA protein assay (Applygen Technologies Inc.). Proteins were mixed with 5× SDS sample loading buffer and incubated at 95°C for 10 min. Then, proteins were separated by SDS–PAGE gel, transferred to PVDF membranes (Millipore), and incubated with appropriate primary antibodies coupled with a horseradish peroxidase-conjugated secondary antibody. Images were visualized using ECL western blotting detection reagents (Tanon).

### Coimmunoprecipitation (Co-IP)

Cells were washed twice in cold PBS and lysed in IP buffer (150 mmol/L NaCl, 25 mmol/L Tris-HCl (pH 7.4), 2.5 mmol/L MgCl_2_, 0.5% NP-40, 0.5 mmol/L EDTA, and 5% glycerol) with protease inhibitors. Cell extracts were incubated with the indicated primary antibodies overnight at 4°C and then with Protein A/G Plus-Agarose (Santa Cruz, United States) at 4°C for 2 h. The beads were washed five times with wash buffer (IP buffer without NP-40), eluted in 2× SDS sample loading buffer, and then subjected to electrophoresis.

### Immunostaining

For α-SMA immunostaining, cells cultured on coverslips were fixed with 4% (v/v) formaldehyde at room temperature for 10 min, permeabilized with 0.5% Triton X-100 at room temperature for 20 min, and blocked with 3% BSA at room temperature for 30 min. The slides were incubated with antibodies against α-SMA at 4°C overnight, followed by staining with Alexa Fluor 488-conjugated anti-mouse antibodies and CoraLite® 594-phalloidin for 2 h at room temperature. Finally, the slides were mounted with medium containing DAPI.

### Luciferase Reporter Assay

For the luciferase reporter assay, MRC5 cells were cotransfected with TOP Flash or FOP Flash luciferase reporter. At 48 h post-transfection, the cells were collected and lysed with lysis buffer. The internal control used was pTK-Renilla. Firefly luciferase and Renilla luciferase activities were quantified using the dual-luciferase reporter assay system according to the manufacturer’s instructions (Promega, Madison, WI).

### Statistical Analysis

Statistical significance was calculated using GraphPad Prism 6 software. Data are representative and/or the mean ± SEM of 3 assays. An unpaired two-sided Student’s t test was used to compare two groups, and one-way ANOVA was used to compare multiple groups unless otherwise indicated. Correlations between groups were determined by Pearson’s correlation test. All experiments were conducted with at least 3 biological replicates. In all cases, *p* < 0.05 was considered statistically significant.

## Results

### TRAF6 Expression Was Downregulated in Lung Fibroblasts From PF Mice and TRAF6 Overexpression Protected Mice Against BLM-Induced PF

**FIGURE 1:**
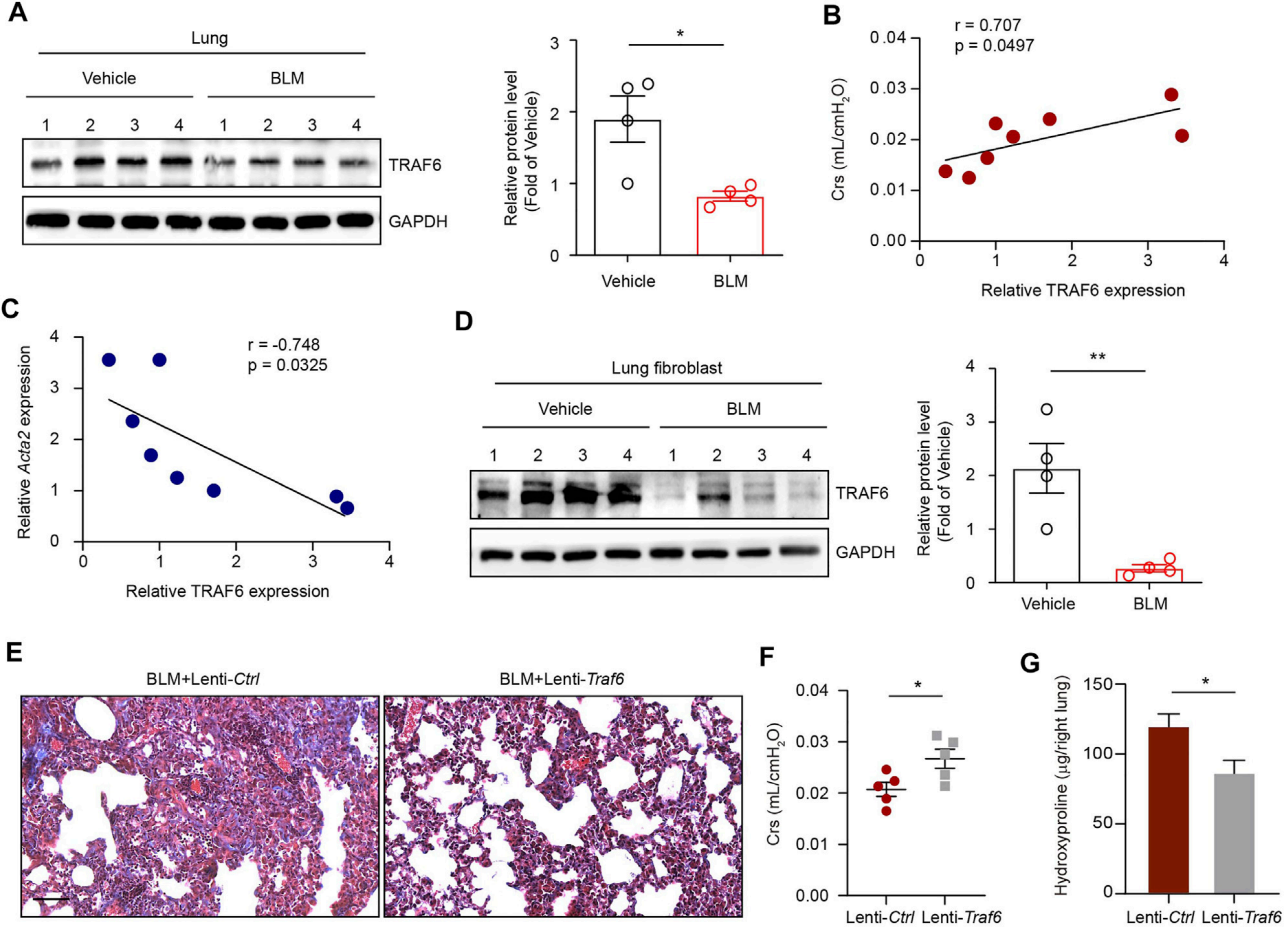
TRAF6 expression in lung fibroblasts is negatively correlated with PF progression. **(A)** Western blot analysis of TRAF6 expression in the lungs of mice following BLM induction. **(B)** Correlation analysis between TRAF6 expression in the lungs of PF mice and respiratory system compliance (Crs) in mice. Each point represents the value of one mouse. Spearman’s rank correlation test was employed to determine statistical significance. **(C)** Correlation analysis between TRAF6 expression and *Acta2* expression in the lungs of PF mice. Each point represents the value of one mouse. Spearman’s rank correlation test was employed to determine statistical significance. **(D)** Western blot analysis of TRAF6 expression in lung fibroblasts isolated from the lungs of mice following BLM induction. **(E)** Masson staining was performed to evaluate fibrotic changes in the indicated mice (Group 1: BLM + Lenti-Ctrl, Group 2: BLM + Lenti-Traf6; n = 5 per group). Scale bars, 100 μm. Crs **(F)** and hydroxyproline content in the right lung **(G)** were assessed to evaluate pulmonary fibrosis and lung function (Group 1: BLM + Lenti-Ctrl, Group 2: BLM + Lenti-Traf6; *n* = 5 per group). Data are representative of 3 independent experiments. Data represent means ± SEM Statistical significance: ^*^ *p* < 0.05 or ^**^ *p* < 0.01.

To address the role of TRAF6 in PF, we first examined TRAF6 expression in the lungs of mice following BLM-induced PF. Western blot analysis showed decreased TRAF6 protein levels in the lungs of PF mice compared to those of controls (Figure 1A). Moreover, decreased TRAF6 in PF lungs was associated with reduced pulmonary function (Figure 1B). The protein expression of TRAF6 was negatively correlated with the mRNA expression of *Acta2* (a marker of myofibroblasts) in fibrotic lungs (Figure 1C). These results prompted us to detect TRAF6 expression in lung fibroblasts, the effector cells in PF progression. Indeed, lower expression of TRAF6 was observed in lung fibroblasts from PF mice than in those from controls (Figure 1D). These findings demonstrate that TRAF6 in lung fibroblasts is negatively correlated with PF development.

To further confirm whether TRAF6 loss contributes to PF development *in vivo*, we instilled TRAF6-overexpressing lentivirus into lung tissue 10 days after BLM challenge. Overexpression of TRAF6 in lung tissue reduced BLM-induced PF changes, as revealed by the reduction in collagen deposition (Figure 1E), the improvement in lung function (Figure 1F), and the decreased hydroxyproline levels (an indicator of collagen content) in BLM mice (Figure 1G). Thus, TRAF6 may play an indispensable protective role in the progression of PF.

### TRAF6 Overexpression Attenuates the Activation of Lung Fibroblasts From PF Mice

**FIGURE 2:**
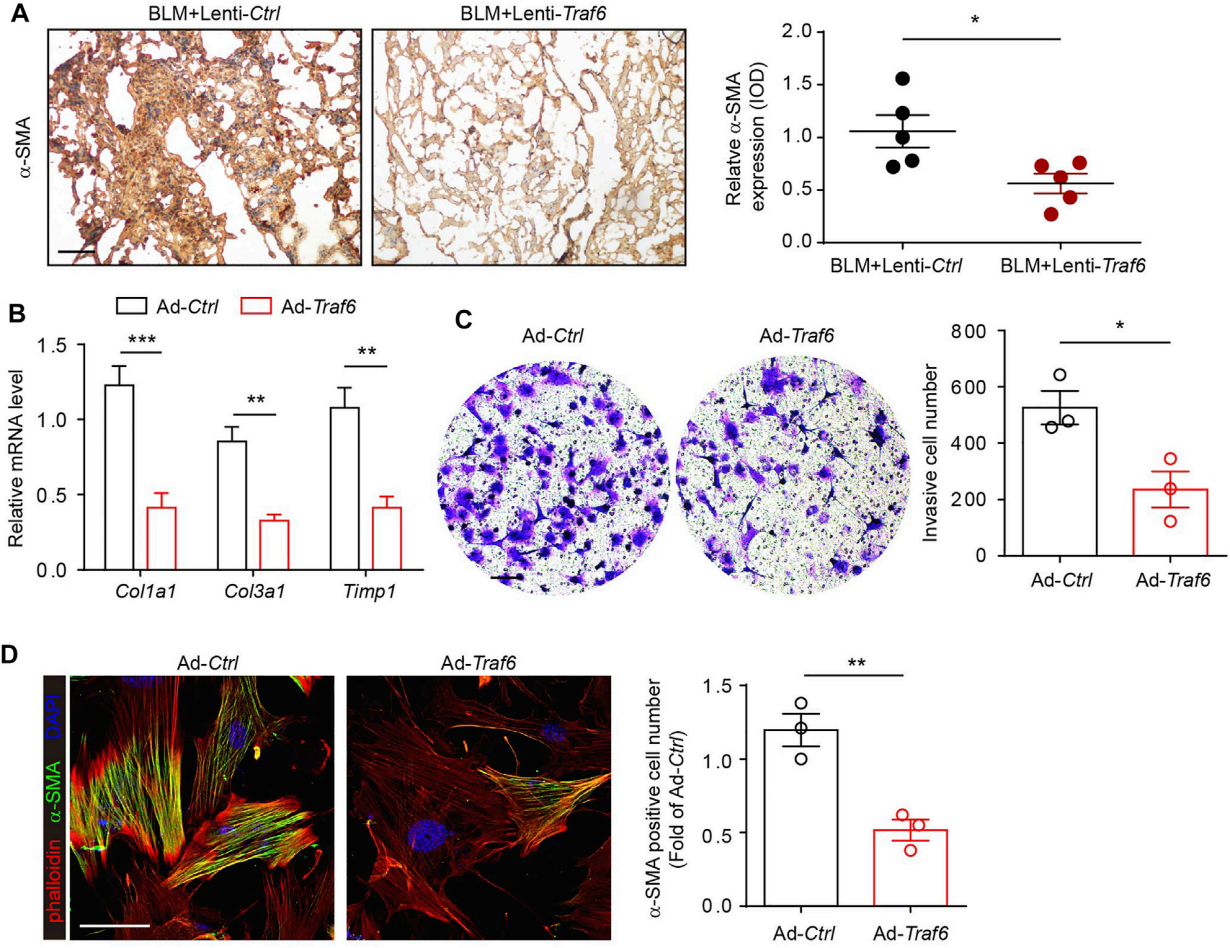
Overexpression of TRAF6 suppresses fibroblast activation. **(A)** IHC staining showing the expression of α-SMA in lung tissues. Representative data and quantified results are shown (Group 1: BLM + Lenti-*Ctrl*, Group 2: BLM + Lenti-*Traf6*; *n* = 5 per group). Scale bar, 100 μm. **(B)** mRNA expression of fibrosis genes in mouse PF lung fibroblasts with or without TRAF6 overexpression. **(C)** Representative images and quantification of the invasion experiment results for mouse PF lung fibroblasts with or without TRAF6 overexpression. Scale bars, 100 μm. **(D)** Representative images and quantification of α-SMA immunostaining in mouse PF fibroblasts with or without TRAF6 overexpression. Scale bars, 50 μm. Data are representative of 3 independent experiments. Data represent means ± SEM Statistical significance: ^*^ *p* < 0.05, ^**^ *p* < 0.01 or ^***^ *p* < 0.001.

We next explored the functional relevance of TRAF6 in fibroblasts during PF progression. IHC staining showed that the expression of α-SMA was reduced in lung tissues from BLM-challenged mice under conditions of TRAF6 overexpression (Figure 2A), suggesting that TRAF6 overexpression suppressed fibroblast activation. To confirm this observation, primary mouse lung fibroblasts isolated from PF mice were transfected with a TRAF6-overexpressing adenovirus. PCR analysis revealed that the expression levels of several fibrosis-related genes and ECM proteins, including *Col1a1*, *Col3a1,* and *Timp1*, were reduced in fibroblasts overexpressing TRAF6 (Figure 2B). The matrigel invasion assay showed that the overexpression of TRAF6 decreased the invasive capacity of lung fibroblasts from PF mice (Figure 2C). In addition, the induction of TRAF6 overexpression in lung fibroblasts derived from PF mice decreased the percentages of α-SMA-positive cells (Figure 2D). Collectively, our data suggest that TRAF6 overexpression reverses the activation of myofibroblasts from PF mice and that TRAF6 deficiency during PF progression promotes the differentiation of lung fibroblasts into myofibroblasts.

### TRAF6 Overexpression Decreases Wnt3a Expression

**FIGURE 3:**
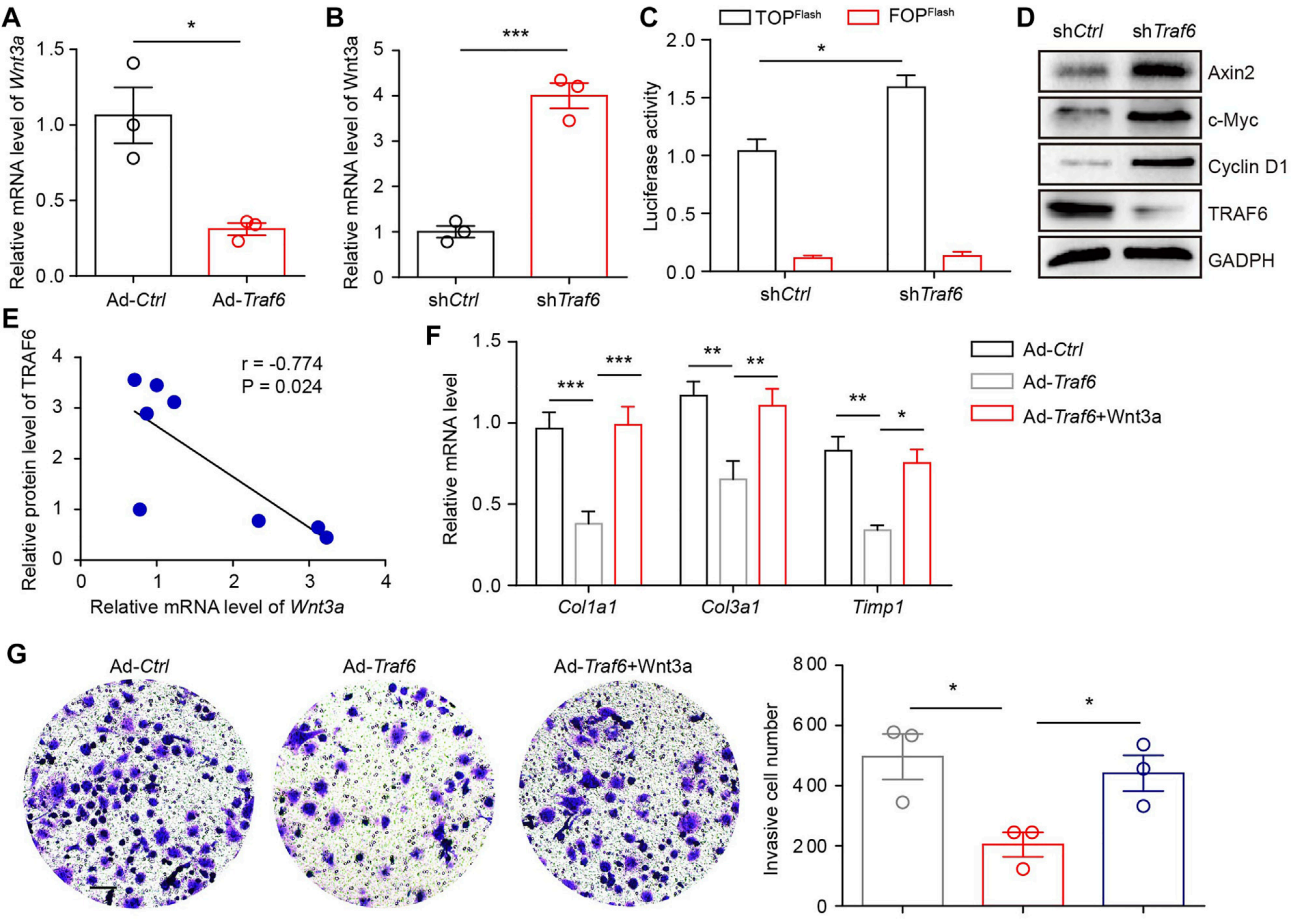
TRAF6 suppresses fibroblast activation by reducing Wnt3a expression. **(A)** mRNA levels of *Wnt3a* as measured by qRT–PCR in TRAF6-overexpressing lung fibroblasts. **(B)** mRNA levels of *Wnt3a* as measured by qRT–PCR in TRAF6-depleted lung fibroblasts. **(C)** Wnt-reporter luciferase activity in PF lung fibroblasts with or without TRAF6 depletion. **(D)** Western blot analysis of β-catenin target gene expression in PF lung fibroblasts with or without TRAF6 depletion. **(E)** Correlation analysis of TRAF6 protein expression and *Wnt3a* mRNA levels in mouse PF lung fibroblasts. **(F)** qRT–PCR analysis of the expression of fibrosis genes in TRAF6-overexpressing mouse lung fibroblasts with or without Wnt3a stimulation. **(G)** Transwell assay for the evaluation of the invasion of TRAF6-overexpressing lung fibroblasts with or without Wnt3a stimulation. Scale bars, 100 μm. Data are representative of 3 independent experiments. Data represent means ± SEM Statistical significance: ^*^ *p* < 0.05, ^**^ *p* < 0.01 or ^***^ *p* < 0.001.

We explored how TRAF6 regulates the activation of lung fibroblasts. A previous study reported that TRAF6 overexpression in cardiac stem cells induced the downregulation of Wnt3a (Cao et al., 2015), which has been implicated in the pathogenesis of PF. We found that the overexpression of TRAF6 in lung fibroblasts from PF mice reduced the mRNA level of *Wnt3a* and that the depletion of TRAF6 in lung fibroblasts from control mice increased the mRNA level of *Wnt3a* (Figures 3A,B). TRAF6 depletion enhanced Wnt-reporter luciferase activity in PF lung fibroblasts (Figure 3C). In addition, western blot analysis demonstrated elevated expression of Wnt/β-catenin target genes in lung fibroblasts with TRAF6 depletion (Figure 3D). Moreover, the protein expression of TRAF6 was negatively correlated with the mRNA expression of *Wnt3a* in fibroblasts from PBS- and BLM-challenged mice (Figure 3E). The decreased expression of fibrosis-related genes caused by TRAF6 overexpression was reversed in lung fibroblasts treated with Wnt3a (Figure 3F). Furthermore, stimulation of lung fibroblasts with Wnt3a enhanced the invasive capacity that was suppressed by TRAF6 overexpression (Figure 3G). These data suggest that TRAF6 regulates fibroblast activation via Wnt3a.

### TRIB3 Is Responsible for the Reduced Expression of TRAF6

**FIGURE 4:**
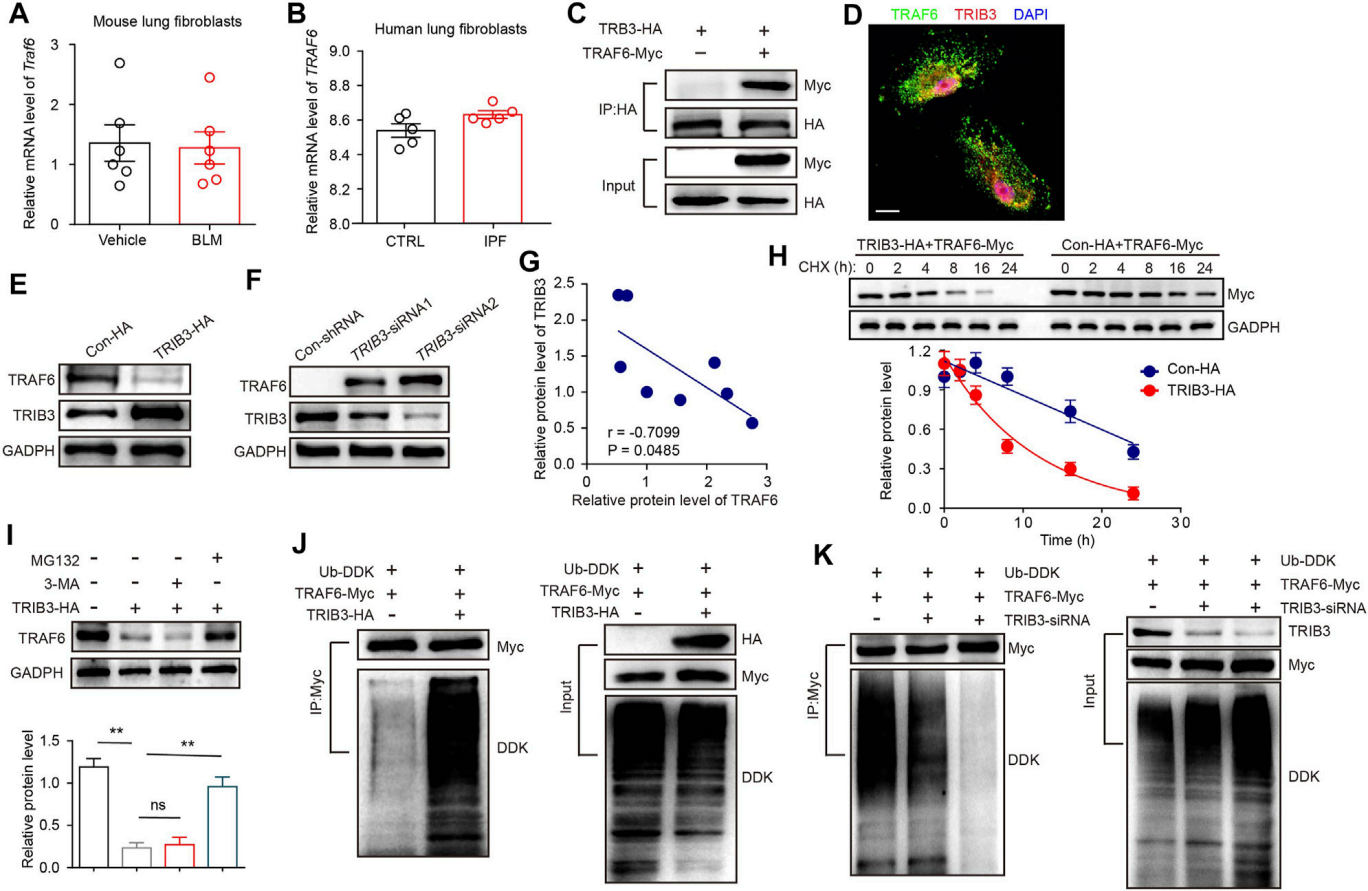
TRIB3 decreases the expression of TRAF6 by promoting its degradation. **(A)** mRNA levels of *Traf6* as measured by qRT–PCR in lung fibroblasts from vehicle- and BLM-challenged mice. **(B)** *TRAF6* mRNA expression in lung fibroblasts of IPF patients compared with normal controls based on the reanalysis of a published dataset (GSE129164). **(C)** The interaction between TRAF6 and TRIB3 was evaluated by co-IP assays. HEK293T cell extracts were subjected to IP with an HA antibody and blotted with a Myc antibody. **(D)** Colocalization of TRAF6 and TRIB3 was evaluated in primary lung fibroblasts. Scale bars, 10 μm. **(E)** Sample immunoblot analyses of the expression of TRAF6 after TRIB3 overexpression in MRC5 cells. **(F)** Sample immunoblot analyses of the expression of TRAF6 after TRIB3 silencing in MRC5 cells. **(G)** Correlation analysis of TRAF6 protein expression and TRIB3 protein expression in mouse PF lung fibroblasts. **(H)** TRAF6 degradation in HEK293T cells with or without TRIB3 overexpression was determined by western blotting. **(I)** Analysis of the TRAF6 degradation pathway. Lung fibroblasts were incubated with CHX (20 μM), CHX plus 3-Methyladenine (3-MA, 5 mM), or MG132 (10 μM) for the indicated times. TRAF6 expression was detected by immunoblotting. **(J)** Immunoblots of ubiquitinated TRAF6 after TRIB3 overexpression. **(K)** Immunoblots of ubiquitinated TRAF6 after TRIB3 deletion. Data are representative of 3 independent experiments. Data represent means ± SEM Statistical significance: ^**^ *p* < 0.01.

We further investigated why TRAF6 expression is downregulated in fibroblasts during PF progression. PCR results showed that *Traf6* mRNA expression was comparable in lung fibroblasts from vehicle- and BLM-challenged mice (Figure 4A). We further analyzed TRAF6 gene expression in lung fibroblasts from IPF patients and healthy controls using a public microarray dataset (GSE129164). No difference in *TRAF6* mRNA expression was found between lung fibroblasts from IPF patients and healthy controls (Figure 4B). These data indicated that the decreased protein expression of TRAF6 in lung fibroblasts during PF progression might result from an alteration in its protein stability. Our group, along with others, has reported that TRIB3, a well-known stress sensor, is involved in regulating the stability of various proteins in the pathogenesis of chronic diseases (Lin et al., 2019; Yu J. M. et al., 2019; Liu S. et al., 2021). We then reasoned that TRIB3 might participate in regulating the altered protein expression of TRAF6. Indeed, an interaction between exogenous TRIB3 and TRAF6 was observed in HEK293T cells, and this was confirmed by the colocalization of TRIB3 and TRAF6 in primary mouse lung fibroblasts (Figures 4C,D). To map the interaction regions of TRAF6 and TRIB3, coIP assays were carried out using serial deletion mutants of either Myc-tagged TRAF6 or HA-tagged TRIB3. TRIB3 interacted with the ΔC domain but not with the ΔN domain of TRAF6 (Supplementary Figure S1A), and TRAF6 interacted with the KD and ΔN domains of TRIB3 (Supplementary Figure S1B). These data indicate that the ΔC domain of TRAF6 and the KD and ΔN domains of TRIB3 are required for the interaction between TRAF6 and TRIB3.

Overexpression of TRIB3 decreased the protein level of TRAF6, and depletion of TRIB3 increased the protein level of TRAF6 (Figures 4E,F). Moreover, the expression of TRAF6 was positively correlated with that of TRIB3 in lung fibroblasts from PBS- and BLM-challenged mice (Figure 4G). Indeed, the overexpression of TRIB3 reduced the half-life of TRAF6 (Figure 4H). Intracellular proteins are degraded by lysosomal autophagy pathways or the ubiquitin–proteasome system (UPS). We found that the UPS inhibitor MG132, but not the autophagy inhibitor 3-MA, reversed the TRIB3 overexpression-induced reduction in TRAF6 expression (Figure 4I). Furthermore, the overexpression and depletion of TRIB3 increased and blocked the ubiquitination of TRAF6, respectively (Figures 4J,K). These data indicate that the protein expression of TRAF6 can be regulated by TRIB3.

**FIGURE 5:**
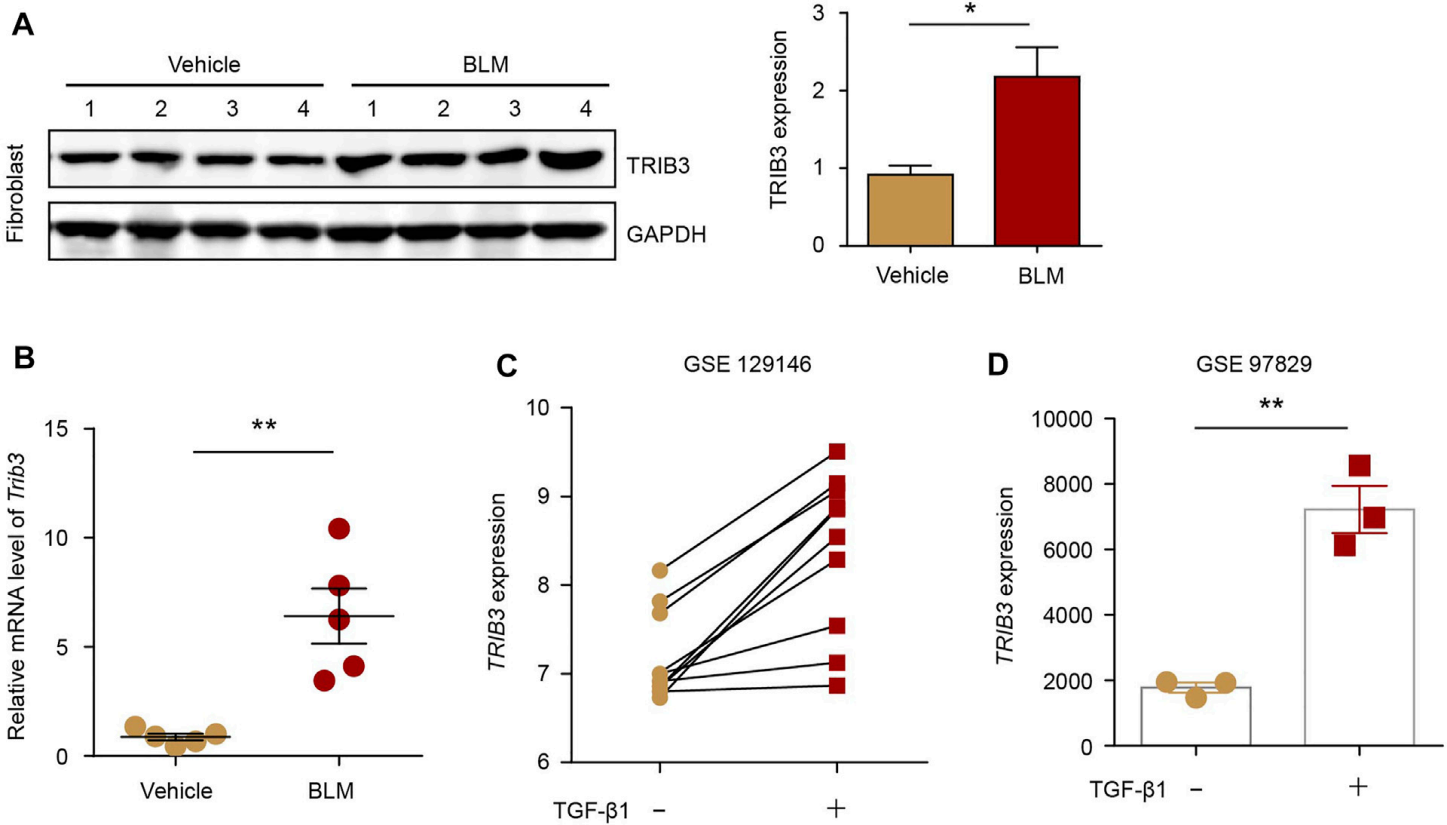
TRIB3 expression is increased in PF lung fibroblasts. **(A)** Western blot analysis of TRIB3 expression in lung fibroblasts isolated from the lungs of mice following BLM induction. **(B)** mRNA levels of *Trib3* as measured by qRT–PCR in lung fibroblasts from vehicle- and BLM-challenged mice. **(C)** *TRIB3* mRNA expression in lung fibroblasts of IPF patients and normal controls treated with or without TGF-β1 based on the reanalysis of a published dataset (GSE129164). **(D)** *TRIB3* mRNA expression in lung fibroblasts treated with or without TGF- β1 based on the reanalysis of a published dataset (GSE97829). Data are representative of 3 independent experiments. Data represent means ± SEM Statistical significance: ^*^ *p* < 0.05 or ^**^ *p* < 0.01.

We then detected the expression of TRIB3 in lung fibroblasts during PF progression. TRIB3 exhibited markedly higher protein and mRNA expression levels in lung fibroblasts from PF mice than in those from control mice (Figures 5A,B). Based on the reanalysis of a published dataset (GSE129164), we found that stimulation of lung fibroblasts of IPF patients and normal controls with TGF-β1 (the principal profibrotic cytokines in lung fibrosis) increased *TRIB3* mRNA expression (Figure 5C). Another dataset also revealed higher *TRIB3* mRNA levels in human lung fibroblasts treated with TGF-β1 than in untreated cells (Figure 5D). Collectively, these data indicate that the enhanced expression of TRIB3 in lung fibroblasts results in a reduction in TRAF6 expression by promoting its ubiquitination and subsequent degradation.

### TRAF6 Is Required for the Role of TRIB3 in Regulating Fibroblast Activation

**FIGURE 6:**
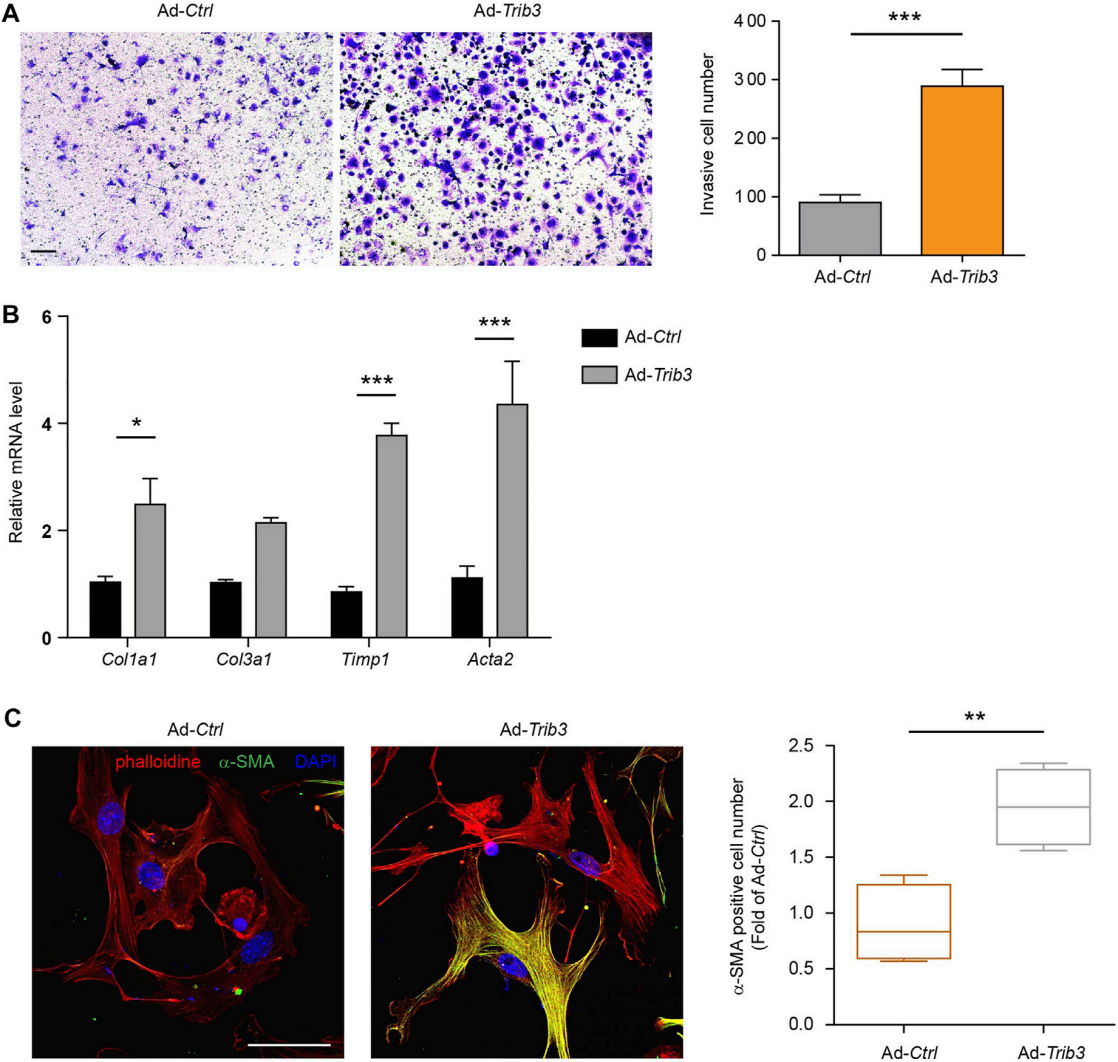
Overexpression of TRIB3 activates lung fibroblasts. **(A)** Representative images and quantification of the invasion experiment results for mouse lung fibroblasts with or without TRIB3 overexpression. Scale bars, 100 μm. **(B)** mRNA expression of fibrosis genes in mouse lung fibroblasts with or without TRIB3 overexpression. **(C)** Representative images and quantification of α-SMA immunostaining in mouse lung fibroblasts with or without TRIB3 overexpression. Scale bars, 50 μm. Data are representative of 3 independent experiments. Data represent means ± SEM Statistical significance: ^*^ *p* < 0.05, ^**^ *p* < 0.01 or ^***^ *p* < 0.001.

**FIGURE 7:**
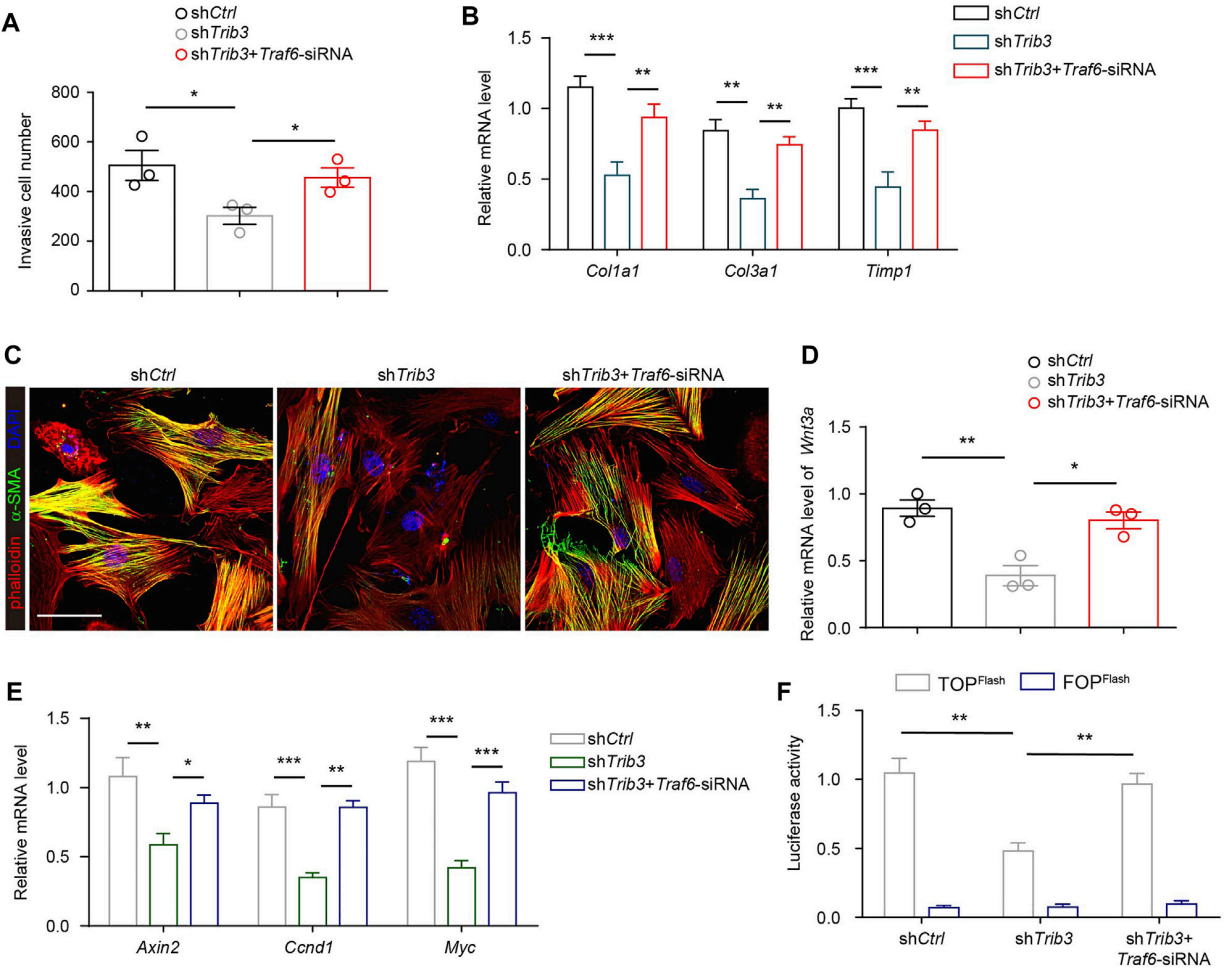
TRIB3 activates lung fibroblasts by reducing TRAF6 expression. **(A)** Transwell assay for the evaluation of invasion of TRIB3-silenced PF lung fibroblasts with or without TRAF6 knockdown. **(B)** mRNA expression of fibrosis genes in TRIB3-silenced PF lung fibroblasts with or without TRAF6 knockdown. **(C)** Representative images of α-SMA immunostaining in TRIB3-silenced PF lung fibroblasts with or without TRAF6 knockdown. Scale bars, 50 μm. **(D)** mRNA expression of *Wnt3a* in TRIB3-silenced PF lung fibroblasts with or without TRAF6 knockdown. **(E)** mRNA expression of the β-catenin target genes in TRIB3-silenced PF lung fibroblasts with or without TRAF6 knockdown. **(F)** Wnt-reporter luciferase activity in TRIB3-silenced PF lung fibroblasts with or without TRAF6 knockdown. Data are representative of 3 independent experiments. Data represent means ± SEM Statistical significance: ^*^ *p* < 0.05, ^**^ *p* < 0.01 or ^***^ *p* < 0.001.

Since the reduction in TRAF6 expression caused by TRIB3 contributes to the differentiation of fibroblasts to myofibroblasts, the above data prompted us to analyze the role of TRIB3 in regulating fibroblast activation. TRIB3 overexpression enhanced the invasive capacity of primary mouse lung fibroblasts (Figure 6A). Much higher levels of fibrosis-related genes were detected in TRIB3-overexpressing fibroblasts than in control fibroblasts (Figure 6B). Immunostaining analysis also indicated that TRIB3 overexpression in lung fibroblasts significantly increased the number of α-SMA-positive cells (Figure 6C), suggesting that TRIB3 induced the differentiation of fibroblasts into myofibroblasts. Consistently, we found that TRIB3 deficiency reversed the activation of myofibroblasts, as indicated by decreased invasive capacity, reduced expression of fibrosis-related genes, and fewer α-SMA-positive cells (Figures 7A–C). However, depletion of TRIB3 failed to inhibit the activation of TRAF6-silenced fibroblasts (Figures 7A–C). In addition, TRIB3 knockdown decreased the expression of *Wnt3a* and Wnt/β-catenin target genes in wild-type PF lung fibroblasts but not in fibroblasts with TRAF6 depletion (Figures 7D,E). Furthermore, TRIB3 depletion failed to reduce Wnt-reporter luciferase activity in lung fibroblasts with TRIB3 knockdown (Figure 7F). These results collectively demonstrate a TRIB3-TRAF6-Wnt3a signaling axis in regulating fibroblast activation.

## Discussion

**FIGURE 8:**
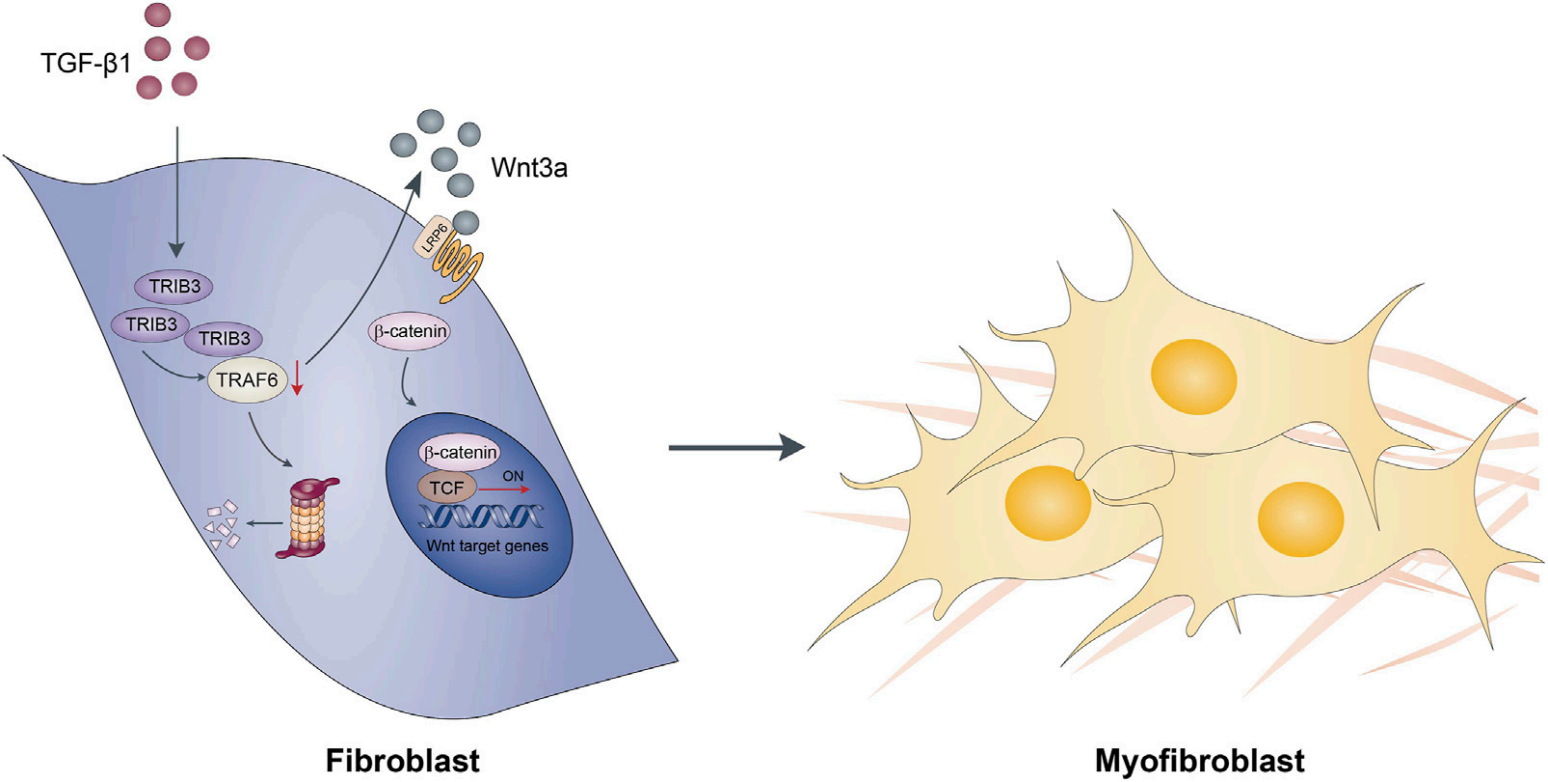
Schematic diagram showing possible mechanisms by which TRAF6 involves in the development of pulmonary fibrosis.

TRAF6, a member of the TRAF family, plays a crucial role in immune signal transduction by acting as an adaptor protein downstream of multiple receptor families, including the interleukin-1 receptor (IL-1R) superfamily, the Toll-like receptor (TLR) family, and the TNFR superfamily. In recent years, TRAF6 has been extensively investigated in tumors, immunity, neurodegenerative diseases, ischemic stroke and osteoporosis (Li et al., 2017; Dou et al., 2018; Muto et al., 2022). Recent studies have also reported that TRAF6 is involved in the pathogenesis of NASH (Wang et al., 2020). They found that depletion of TRAF6 attenuated liver fibrosis in diet-induced NASH models, supporting a role for TRAF6 in promoting liver fibrosis. However, the role of TRAF6 in lung fibrosis remains unknown. In this study, we found that TRAF6 was downregulated in fibroblasts within the lung during the course of fibrotic processes. TRAF6 overexpression in fibroblasts from PF mice suppressed the activation of these cells. Furthermore, TRAF6 overexpression protected mice from BLM-induced lung fibrosis, with a decrease in the number of activated fibroblasts, also called myofibroblasts. Our data indicate that the downregulation of TRAF6 expression in fibroblasts contributes to PF progression by inducing the activation of those cells (Figure 8). This study selectively focused on the role of TRAF6 in fibroblasts. Given that TRAF6 is an intracellular protein widely expressed in epithelial cells, endothelial cells, and macrophages, future studies are needed to investigate the expression and role of TRAF6 in those cells during pulmonary fibrosis.

TRIB3, a stress sensor, is involved in the pathogenesis of various diseases, including obesity, diabetes, and tumors (Du et al., 2003; Oberkofler et al., 2010; Lin et al., 2019). Accumulating evidence has widely demonstrated that TRIB3 plays a vital role in organ fibrogenesis (Wang et al., 2014; Tomcik et al., 2016; Zhang et al., 2020). Our previous study, along with others, reported the key role of TRIB3 in promoting PF (Yu W. et al., 2019; Liu S. et al., 2021). We found that TRIB3 was substantially upregulated in alveolar macrophages (AMs) from patients with PF, inducing the profibrotic phenotype of AMs. Moreover, genetic knockout of TRIB3 specifically in AMs suppressed BLM-induced fibrotic changes in the lung. Another study found a central regulatory role of TRIB3 in fibroblast activation by stimulating canonical TGF-β1/Smad signaling in systemic sclerosis (Tomcik et al., 2016). Here, we found that elevated TRIB3 expression in fibroblasts from PF mice contributes to PF progression by enhancing the expression of Wnt3a, a crucial factor in the regulation of fibroblast activation (Li X. et al., 2020; Liu T. et al., 2021). Collectively, these data support that TRIB3 exerts profibrotic roles in different cell types during PF progression, demonstrating its potential value in the clinical treatment of PF.

As a member of the pseudokinase family, TRIB3 was found to regulate diverse processes in the progression of various diseases by interacting with functional proteins. For instance, the interaction of TRIB3 and the protein kinase GSK-3β interferes with the binding of GSK-3β to the E3 ligase UHRF1, thereby inhibiting the degradation of GSK-3β and leading to the profibrotic phenotype of Ams (Liu S. et al., 2021). TRIB3 interacted with MYC to suppress E3 ubiquitin ligase UBE3B-mediated MYC degradation, which induced the enhanced expression of MYC, causing the proliferation of lymphoma cells (Li K. et al., 2020). In the current study, the interaction of TRIB3 with TRAF6 in fibroblasts resulted in reduced TRAF6 expression. Intracellular proteins are degraded by the UPS or lysosomal autophagy pathway. We found that the UPS inhibitor MG132, but not the autophagy inhibitor 3-MA, reversed the TRIB3 overexpression-induced reduction in TRAF6 expression, indicating that the decrease in the protein level of TRAF6 caused by TRIB3 was related to its increased degradation through the UPS. This was consistent with previous studies showing that TRAF6 is degraded via the UPS (Li et al., 2014; Kim et al., 2017). Indeed, we observed increased ubiquitination and degradation of TRAF6 in TRIB3-overexpressing cells. We presumed that TRIB3, acting as a scaffolding protein, interacts with TRAF6 to recruit certain E3 ligases, thereby causing the ubiquitination and subsequent degradation of TRAF6. Obviously, further investigation of which kind of E3 ligase accounts for TRIB3 regulation of TRAF6 expression is warranted.

Increasing lines of evidence demonstrate that the aberrant activation of Wnt signaling is involved in the pathogenesis of PF (Morrisey, 2003; Skronska-Wasek et al., 2018). Attenuation of Wnt/β-catenin signaling can limit the development of fibrosis in mice. Wnt3a, the canonical ligand, is known to induce the activation of fibroblasts to myofibroblasts (Li X. et al., 2020). We found that depletion of TRAF6 in lung fibroblasts from control mice increased the expression of Wnt3a and Wnt/β-catenin target genes. A negative correlation was found between the expression of TRAF6 and Wnt3a in fibroblasts from PBS- and BLM-challenged mice. This was consistent with a prior study showing that TRAF6 overexpression induced the downregulation of Wnt3a in c-kit^+^ cardiac stem cells. We also found that Wnt3a treatment reversed the suppressed activation of fibroblasts caused by TRAF6 overexpression. These results indicated that reduced expression of TRAF6 in lung fibroblasts induces the activation of fibroblasts by enhancing the expression of Wnt3a during PF progression. Thus, our work provides insight into the regulatory role of TRAF6 in Wnt3a expression in fibroblasts. Further investigation is necessary to determine the molecular mechanism by which TRAF6 controls Wnt3a expression.

Our findings revealed that the reduced expression of TRAF6 caused by TRIB3 overexpression in lung fibroblasts contributes to the progression of PF through upregulation of Wnt3a expression, which drives fibroblast differentiation into myofibroblasts. Accordingly, genetically enhancing TRAF6 expression or inhibiting the TRIB3-TRAF6 interaction may represent a novel therapeutic strategy for PF and other fibroproliferative lung diseases.

## Data Availability

The original contributions presented in the study are included in the article/Supplementary Material, further inquiries can be directed to the corresponding author.
