# Supplementary material for: TRAF6 Suppresses the Development of Pulmonary Fibrosis by Attenuating the Activation of Fibroblasts
Source: Front Pharmacol. 2022 May 20;13:911945. doi: 10.3389/fphar.2022.911945 (PMC9163739; doi:10.3389/fphar.2022.911945)
Supplement: Supplementary file 1 [file DataSheet1.docx]

**Supplementary Information**

**Inventory of Supplementary Information**

Supplementary Table 1

Supplementary Figure S1

**Supplementary Table**

**Supplementary Table 1.** The primer sequences used for qRT-PCR.

| Gene |  | Primer Sequence (5’-3’) |
| --- | --- | --- |
| *Col1a1* | Forward | CCTCAGGGTATTGCTGGACAAC |
|  | Reverse | CAGAAGGACCTTGTTTGCCAGG |
| *Col3a1* | Forward | GACCAAAAGGTGATGCTGGACAG |
|  | Reverse | CAAGACCTCGTGCTCCAGTTAG |
| *Timp1*  *Wnt3a*  *Traf6*  *Trib3*  *Acta2*  *Axin2*  *Ccnd1*  *Myc*  *Gapdh* | Forward  Reverse  Forward  Reverse  Forward  Reverse  Forward  Reverse  Forward  Reverse  Forward  Reverse  Forward  Reverse  Forward  Reverse  Forward  Reverse | TCTTGGTTCCCTGGCGTACTCT  GTGAGTGTCACTCTCCAGTTTGC  AACTGCACCACCGTCAGCAACA  AGCGTGTCACTGCGAAAGCTAC  TTTCCCTGACGGTAAAGTGCCC  ACCTGGCACTTCTGGAAAGGAC  CTGCGTCGCTTTGTCTTCAGCA  CTGAGTATCTCTGGTCCCACGT  GAGATTGTGCCAGTGCTGGTGT  GTGACAGTTCCTGTCCCATCAG  ATGGAGTCCCTCCTTACCGCAT  GTTCCACAGGCGTCATCTCCTT  GCAGAAGGAGATTGTGCCATCC  AGGAAGCGGTCCAGGTAGTTCA  TCGCTGCTGTCCTCCGAGTCC  GGTTTGCCTCTTCTCCACAGAC  CATCACTGCCACCCAGAAGACTG  ATGCCAGTGAGCTTCCCGTTCAG |

**Supplementary Figures**


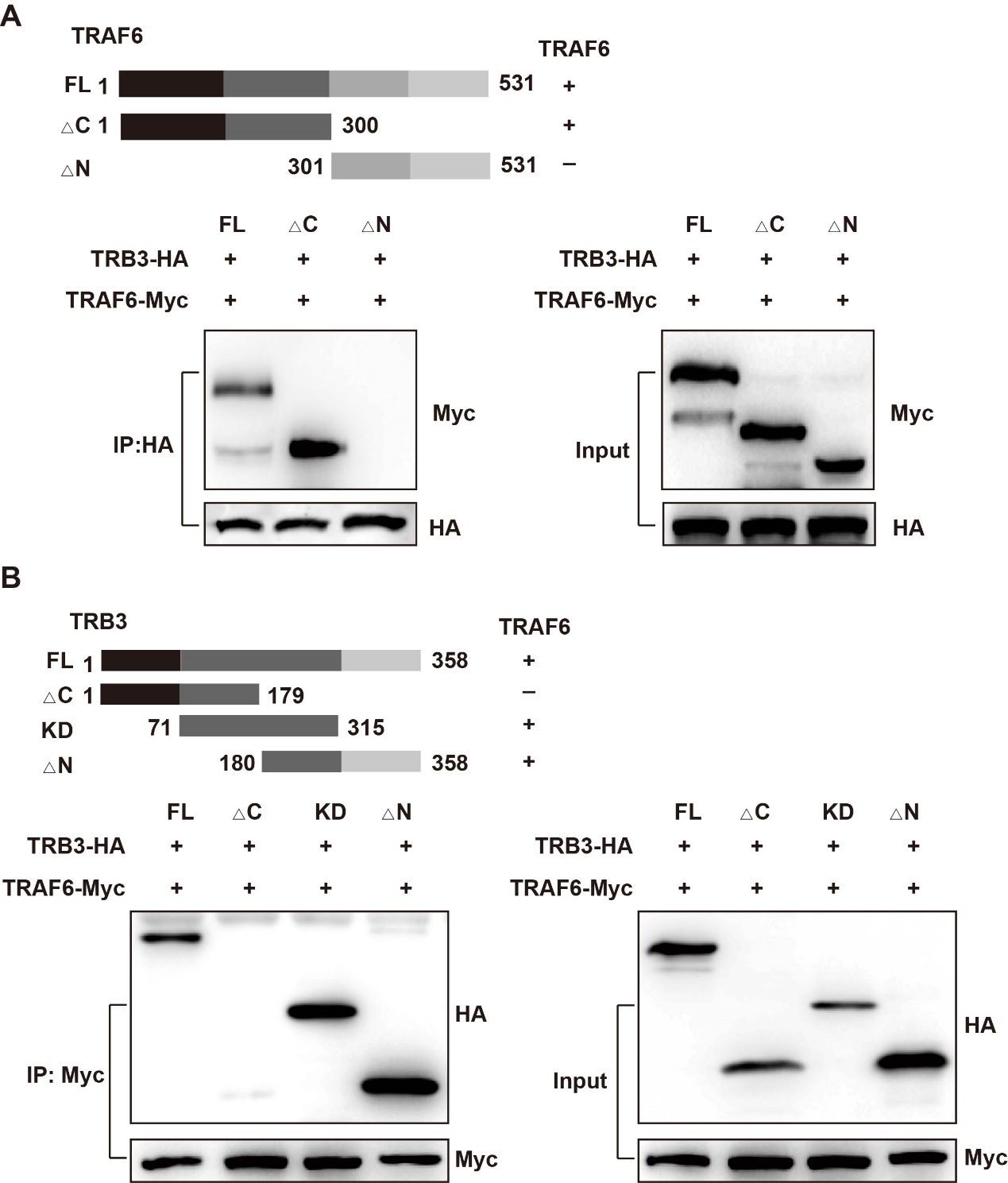


**Supplementary Figure S1** TRAF6 interacts with TRIB3. **(A)** Mapping of TRAF6 regions involved in TRIB3 binding. Schematic diagram of TRAF6 and TRAF6 deletion mutants (top). Sample blot showing the interaction of each mutant with TRIB3, marked as + or -. **(B)** Mapping of TRIB3 regions involved in TRAF6 binding. Schematic diagram of TRIB3 and TRIB3 deletion mutants (top). Sample blots showing the interaction of each mutant with TRAF6, marked as + or -.
